# Supplementary material for: Metabolic phenotyping of BMI to characterize cardiometabolic risk: evidence from large population-based cohorts
Source: Nat Commun. 2023 Oct 7;14:6280. doi: 10.1038/s41467-023-41963-7 (PMC10560260; doi:10.1038/s41467-023-41963-7)
Supplement: Supplementary file 1 — Supplementary Information [file 41467_2023_41963_MOESM1_ESM.pdf]

Metabolic phenotyping of BMI to characterize cardiometabolic risk: evidence from large population-based cohorts

Habtamu B Beyene[1,2,3,4]*, Corey Giles[1,3,4]*, Kevin Huynh[1,3,4], Tingting Wang[1,3,4], Michelle Cinel[1], Natalie A Mellett[1], Gavriel Olshansky[1], Thomas G Meikle[1,3], Gerald F Watts [5,6], Joseph Hung[5], Jennie Hui[7,8], Gemma Cadby[8], John Beilby[7], John Blangero[9], Eric K Moses[10,11], Jonathan E Shaw[1,2], Dianna J Magliano[1,2,] **, Peter J Meikle[1,2,3,4]**

[1]Baker Heart and Diabetes Institute, Melbourne, Australia.
[2]Faculty of Medicine, Nursing and Health Sciences, Monash University, Melbourne, Australia.
[3]Baker Department of Cardiovascular Research Translation and Implementation, La Trobe University, Melbourne, Australia.
[4]Baker Department of Cardiometabolic Health, Melbourne University, Melbourne, Australia.
[5]School of Medicine, University of Western Australia, Perth, Australia.
[6]Lipid Disorders Clinic, Department of Cardiology, Royal Perth Hospital, Perth, Australia.
[7]PathWest Laboratory Medicine of Western Australia, Nedlands, Western Australia; School of Biomedical Sciences, University of Western Australia.
[8]School of Population and Global Health, University of Western Australia, Crawley, Western Australia.
[9]South Texas Diabetes and Obesity Institute, The University of Texas Rio Grande Valley, Brownsville, Texas, USA.
[10]School of Biomedical Sciences, University of Western Australia, Crawley, Western Australia, Australia. [11]Menzies Institute for Medical Research, University of Tasmania, Hobart, Tasmania, Australia.

*Co-first authors

**Co-senior and corresponding authors

Supplementary Table 1: Baseline characteristics of the AusDiab participants stratified by Type 2 Diabetes and CVD status

Characteristic	Cross-sectional T2DM analysis			Longitudinal T2DM analysis		
	**Controls (NGT)**	**Prevalent T2DM**	**p-value**	**Controls (NGT)**	**Incident T2DM**	**p-value**
n	7,733	395		5,354	218	
Age (years)[a]	48.9 (13.7)	61.6 (13.2)	**6.25E-58**	50.7 (12.6)	55.7 (12.0)	**9.23E-09**
Sex, n (%men)[b]	3,350 (43.3)	196 (49.6)	**1.59E-02**	2,409 (45.0)	112 (51.4)	7.41E-02
BMI (kg/m^2)[a]	26.2 (4.5)	28.9 (6.1)	**2.07E-28**	26.6 (4.6)	29.5 (5.6)	**5.97E-13**
WC (cm)[a]	88.5 (13.0)	100.0 (14.9)	**1.21E-43**	89.6 (13.2)	91.8 (14.5)	**6.62E-17**
Cholesterol (mmol/L)[a]	5.6 (1.1)	5.9 (1.1)	**2.96E-08**	5.7(1.1)	5.9 (1.1)	**2.37E-04**
HDL-C (mmol/L)[a]	1.45 (0.38)	1.35 (0.36)	**1.34E-11**	1.45 (0.38)	1.33 (0.40)	**1.13E-05**
Triglycerides (mmol/L)[c]	1.17 (0.9)	1.94 (1.6)	**4.36E-55**	1.21 (0.90)	1.70 (1.26)	**9.03E-19**
SBP (mmHg)[a]	126.0 (17.4)	145.0 (19.0)	**6.11E-58**	127.0 (17.2)	137.0 (17.6)	**1.97E-12**
DBP (mmHg)[a]	69.0 (11.4)	76.0 (11.8)	**4.69E-24**	69.8 (11.5)	74.7 (11.8)	**5.84E-09**
FBG (mmol/L)[a]	5.3 (0.4)	7.1 (1.9)	**1.43E-60**	5.4 (0.5	6.0 (0.6)	**1.86E-34**
2h-PLG (mmol/L)[a]	5.5 (1.1)	12.3 (3.8)	**1.07E-126**	5.8 (1.5)	7.9 (1.8)	**3.02E-41**
HbA1C (%)[a]	5.1 (0.3)	5.9 (1.0)	**1.38E-46**	5.1 (0.3)	5.4 (0.3)	**2.11E-37**
Current smoking, n (%)[b]	1280 (16.5)	54 (13.7)	1.19E-01	584 (10.9)	35 (16.1)	**2.38E-02**
BP treatment, n (%)[b]	824 (10.7)	148 (37.5)	**3.37E-57**	655 (12.2)	62 (28.4)	**5.13E-12**
Lipid lowering medication, n (%)[b]	446 (5.8)	66 (16.7)	**6.45E-18**	371 (6.9)	35 (16.1)	**7.47E-07**
	**Cross sectional CVD analysis**			**Longitudinal CVE analysis**		
	**Control**	**Prevalent CVD**	**p-value**	**Non-events**	**Incident CVE**	**p-value**
n	9690	577		8,136	414	
Age (years)[a]	50.4 (13.9)	66.6 (11.5)	**1.88E-140**	50.2 (13.1)	67.2 (12.1)	**2.99E-142**
Sex, n (%men)[b]	4,279 (44.2)	339 (58.8)	**1.02E-11**	3506 (43.1)	390 (66.1)	**2.89E-27**
BMI (kg/m^2)[a]	26.9 (4.9)	27.9 (4.8)	**2.89E-06**	26.9 (4.9)	27.6 (4.4)	**2.79E-04**
WC (cm)[a]	90.4 (13.7)	96.6 (13.3)	**4.44E-25**	90.2 (13.7)	96.8 (12.4)	**3.14E-32**
Cholesterol (mmol/L)[a]	5.7 (1.1)	5.5 (1.1)	**1.42E-04**	5.7(1.1)	5.8 (1.1)	**1.10E-02**
HDL-C (mmol/L)[a]	1.45 (0.38)	1.35 (0.36)	**6.24E-08**	1.44 (0.38)	1.32 (0.37)	**1.33E-13**
Triglycerides (mmol/L)[c]	1.26 (1.0)	1.43 (1.1)	**1.93E-06**	1.23 (0.99)	1.52 (1.14)	**2.98E-08**
SBP (mmHg)[a]	129.0 (18.3)	140.0 (20.9)	**6.01E-34**	128.0 (17.7)	144.0 (20.4)	**9.17E-62**
DBP (mmHg)[a]	70.0 (11.7)	72.4 (11.9)	**3.81E-06**	69.9 (11.6)	74.6 (12.1)	**1.85E-18**
FBG (mmol/L)[a]	5.5 (1.0)	6.1 (1.9)	**2.30E-13**	5.5 (1.0)	6.0 (1.6)	**1.91E-13**
2h-PLG (mmol/L)[a]	6.2 (2.2)	7.2 (2.9)	**4.88E-14**	6.2 (2.1)	7.3 (3.0)	**9.34E-16**
HbA1C (%)[a]	5.2 (0.5)	5.6 (1.0)	**1.95E-17**	5.2 (0.5)	5.5 (0.9)	**1.07E-19**
Diabetes, n (%)[b]	567 (5.9)	114 (19.8)	**2.23E-38**	436 (5.4)	108 (18.3)	**1.08E-17**
Current smoking, n (%)[b]	1,538 (15.9)	77 (13.3)	1.19E-01	1153 (14.2)	84 (14.3)	9.90E-01
BP treatment, n (%)[b]	1,292 (13.3)	275 (47.7)	**2.45E-109**	655 (12.2)	62 (28.4)	**5.13E-12**
Lipid lowering medication, n (%)[b]	643 (6.6)	228 (37.1)	**5.45E-143**	371 (6.9)	35 (16.1)	**7.47E-07**

[a]Values in the table expressed as mean (±SD) for continuous variables followed by independent Student's t-tests. [b]Pearson's Chi-squared test for dichotomous variables. [c]Values expressed in median (IQR). Significant p-values (two-sided) <0.05 are shown in bold.

Supplementary Table 2: Baseline characteristics of BHS participants stratified by CVD

Characteristics	Control	Prevalent CVD		Non-events	Incident CVE	
n	**4,254**	**238**	**p-value**	**3,970**	**284**	**p-value**
Age (years)[a]	49.7 (17.0)	71.0 (9.5)	**4.51E-103**	48.2 (16.5)	68.8 (11.4)	**2.67E-105**
Sex, n (%men)[b]	1,826 (42.9)	150 (63.0)	**1.83E-09**	1,666 (42.3)	160 (51.1)	**2.85E-03**
BMI (kg/m^2)[a]	26.0 (4.2)	27.2 (4.0)	**1.11E-05**	25.9 (4.2)	27.2 (4.0)	**6.77E-07**
WC (cm)[a]	85.7 (12.6)	94.2 (11.8)	**1.14E-21**	85.1 (12.5)	94.2 (12.8)	**5.70E-18**
Cholesterol (mmol/L)[a]	5.6 (1.1)	6.0 (1.2)	**5.94E-13**	5.5 (1.1)	6.2 (1.1)	**5.21E-20**
HDL-C (mmol/L)[a]	1.40 (0.39)	1.26 (0.45)	**3.24E-06**	1.41 (0.39)	1.29 (0.41)	**7.25E-07**
Triglycerides (mmol/L)[c]	1.0 (0.8)	1.5 (1.1)	**6.25E-10**	1.0 (0.8)	1.4 (1.1)	**2.05E-13**
SBP (mmHg)[a]	124.0 (17.8)	135.0 (18.0)	**3.53E-18**	123.0 (17.3)	138.0 (17.2)	**2.30E-41**
DBP (mmHg)[a]	74.6 (10.5)	74.8 (12.4)	8.05E-01	74.4 (10.4)	77.5 (11.6)	**3.84E-06**
Diabetes, n (%)[a]	133 (3.1)	29 (12.2)	**1.12E-12**	94 (2.4)	39 (12.4)	**3.35E-22**
Current smoking, n (%)[b]	592 (13.9)	14 (5.8)	**9.18E-04**	577 (14.1)	35 (11.2)	**3.47E-02**
Lipid lowering medication, n (%)[b]	67 (1.6)	41 (17.2)	**1.15E-51**	58 (1.5)	9 (2.9)	9.22E-02

[a]Values in the table expressed as mean (±SD) for continuous variables followed by independent Student's t-tests.
[b]Pearson's Chi-squared test for dichotomous variables.
[c]Values expressed in median (IQR).
Significant p-values (two-sided) <0.05 are shown in bold.

Supplementary Table 3: Regularized linear models for the prediction of BMI

**Predicting BMI from lipidomic data (including age and sex)**			**Predicting BMI from clinical risk factors (adjusted for age, sex, total cholesterol, HDL-C and triglycerides)**		**Predicting BMI from common cardiometabolic risk factors, CMRs (age, sex, total cholesterol, HDL-C and triglycerides, FBG, 2h-PLG, Hba1c, HOMA-IR, SBP, DBP)**	
**Model**	**Percent variance explained (R^2)**		**Percent variance explained (R^2)**		**Percent variance explained (R^2)**	
	**AusDiab**	**Validation (BHS)**	**AusDiab**	**Validation (BHS)**	**AusDiab**	**Validation (BHS)***
Ridge	60.4	52.1	15.6	10.4	31.6	31.2
Elastic net	60.8	52.2	15.5	10.0	31.6	31.1
LASSO	60.9	51.9	15.6	10.2	31.5	31.2
Penalty parameters	Ridge (lambda average = 0.094)		Ridge (lambda average= 0.1618276)		Ridge (lambda average= 0.01484968)	
	Lasso (lambda average= 0.007371657)		Lasso (lambda average = 0.00556216)		Lasso (lambda average = 0.001)	
	Elastic net (lambda average= 0.02254397, alpha = 0.3)		Elastic net (lambda average=0.01275466, alpha = 0.3)		Elastic net (lambda average= 0.005336699, alpha = 0.3)	

*2h-PLG and Hba1c not available in the BHS model

Supplementary Table 4: Association of cardiometabolic risk factors with metabolic discordant groups in the AusDiab participants

Risk factors	Q5 relative to Q1 (Covariates: age, sex and BMI)[a]		Q5 relative to Q1 (Covariates: age, sex, BMI, total cholesterol, HDL-C and triglycerides)[b]	
	Fold difference (95% CI)	p-value	Fold difference (95% CI)	p-value
Cholesterol	1.05 (1.04–1.07)	**2.13E-13**	1.04 (1.02–1.05)	**4.79E-10**
HDL-C	0.90 (0.88–0.92)	**8.71E-93**	0.92 (0.90–0.94)	**3.99E-58**
Triglycerides	1.52 (1.45–1.59)	**6.73E-121**	1.22 (1.17–1.28)	**4.60E-39**
FBG	1.02 (1.01–1.03)	**1.97E-06**	1.01(1.00–1.02)	1.88E-01
2h-PLG	1.17 (1.15–1.19)	**1.19E-62**	1.14 (1.12–1.16)	**6.59E-39**
HbA1c	1.01 (1.01–1.01)	**7.60E-06**	1.00 (1.00–1.01)	3.18E-01
HOMA-IR	1.59 (1.50–1.68)	**1.69E-55**	1.47 (1.38–1.56)	**5.03E-36**
SBP	1.04 (1.02–1.05)	**4.97E-10**	1.03 (1.02–1.04)	**2.25E-07**
DBP	1.03 (1.02–1.05)	**1.67E-07**	1.03 (1.02–1.04)	**2.27E-05**

[a]Linear regression analysis between the two most mBMI discordant groups (predictor)and cardiometabolic risk factors (outcome) adjusting for age, sex and BMI.
[b]Model adjusted for clinical lipids on top of the covariates in model a.
CI = confidence interval. No adjustment for multiple comparisons was made.
Significant p-values (two-sided) <0.05) are shown in bold.

Supplementary Table 5: Risk of T2DM across quintiles of mBMIΔ in the AusDiab participants.

mBMIΔ	Newly diagnosed prevalent T2DM (n=395 cases versus 7,733 NGT)		5-year incident T2DM (n=218 cases versus 5354 controls)	
	Odds ratio (95% CI)[a]	p-value	Odds ratio (95% CI)[a]	p-value
Q1 (reference)	-	-	-	-
Q2	1.5 (1.01, 2.3)	**4.42E-02**	1.3 (0.7, 2.2)	3.91E-01
Q3	2.0 (1.4, 3.0)	**6.42E-04**	2.3 (1.4, 3.8)	**8.76E-04**
Q4	2.5 (1.7, 3.7)	**3.65E-06**	2.3 (1.4, 3.8)	**1.06E-03**
Q5	4.5 (3.1, 6.6)	**1.48E-15**	2.5 (1.5, 4.1)	**2.45E-04**
	Odds ratio (95% CI)[b]	p-value	Odds ratio (95% CI)[b]	p-value
Q1 (reference)	-	-	-	-
Q2	1.4 (0.9, 2.2)	1.14E-01	0.9 (0.5, 1.6)	6.98E-01
Q3	1.7 (1.1, 2.5)	**1.35E-02**	2.0 (1.2, 3.2)	**6.16E-03**
Q4	2.1 (1.4,3.2)	**1.66E-04**	1.7 (1.01, 2.7)	**4.62E-02**
Q5	3.6 (2.5, 5.3)	**6.07E-11**	2.0 (1.2, 3.2)	**5.96E-03**

[a]Logistic regression between mBMIΔ quintile and T2DM (prevalent or incident) adjusting for age, sex and BMI

[b]Logistic regression between mBMIΔ quintile and T2DM (prevalent or incident) adjusting for age, sex and BMI plus, clinical lipids (total cholesterol, HDL-C, and triglycerides), smoking status, and diabetes history. No adjustment for multiple comparisons was made. CI = confidence interval

T2DM, Type 2 diabetes mellitus

Significant p-values (two-sided) <0.05 are shown in bold.

Supplementary Table 6: Risk of prediabetes (prevalent and incident) across quintiles of mBMIΔ in the AusDiab participants.

mBMIΔ	Prevalent prediabetes (n=1920 cases versus 7,733 NGT)		5-year incident prediabetes (n=417 cases versus 4023 NGT controls)	
	Odds ratio (95% CI)[a]	p-value	Odds ratio (95% CI)[a]	p-value
Q1 (reference)	-	-	-	-
Q2	1.3 (1.1, 1.6)	**3.16E-03**	1.1 (0.8, 1.6)	4.42E-01
Q3	1.7 (1.4, 2.0)	**3.21E-08**	1.3 (0.9, 1.8)	1.74E-01
Q4	2.0 (1.7, 2.4)	**6.94E-14**	1.6 (1.1, 2.3)	**5.92E-03**
Q5	3.0 (2.5, 3.5)	**1.54E-33**	2.5 (1.8, 3.5)	**3.67E-08**
	Odds ratio (95% CI)[b]	p-value	Odds ratio (95% CI)[b]	p-value
Q1 (reference)	-	-	-	-
Q2	1.3 (1.1, 1.6)	**9.19E-03**	1.1 (0.8, 1.6)	5.37E-01
Q3	1.6 (1.3, 1.9)	**1.66E-06**	1.2 (0.8, 1.7)	3.47E-01
Q4	1.9 (1.5, 2.2)	**5.68E-11**	1.5 (1.0, 2.1)	**3.44E-02**
Q5	2.7 (2.2, 3.2)	**8.11E-26**	2.2 (1.5, 3.0)	**9.46E-06**

[a]Logistic regression between mBMIΔ quintile and prediabetes (prevalent or incident) adjusting for age, sex and BMI and odds ratios computed.
[b]Logistic regression between mBMIΔ quintile and prediabetes (prevalent or incident) adjusting for age, sex and BMI plus, clinical lipids (total cholesterol, HDL-C, and triglycerides) and smoking status.
No adjustment for multiple comparisons was made. CI = confidence interval
Significant p-values (two-sided) <0.05 are shown in bold.

Supplementary Table 7: Independent association between quintiles of mBMIΔ with isolated IGT and IFG in the AusDiab participants.

**mBMIΔ**	**Isolated IGT (n=988 cases versus 7,733 NGT)**		**Isolated IFG (n=613 cases versus 7,733 NGT)**	
	**Odds ratio (95% CI)[a]**	**p-value**	**Odds ratio (95% CI)[b]**	**p-value**
Q1 (reference)	-	-	-	-
Q2	1.3 (1.0, 1.6)	6.05E-02	1.3 (1.0, 1.8)	5.98E-02
Q3	1.5 (1.2, 2.0)	**7.59E-04**	1.4 (1.04, 1.9)	**2.66E-02**
Q4	1.9 (1.5, 2.4)	**4.17E-07**	1.6 (1.2, 2.1)	**2.95E-03**
Q5	2.4 (1.9, 3.0)	**1.70E-12**	2.2 (1.6, 2.9)	**2.73E-07**

[a]Logistic regression between mBMIΔ quintile and IGT adjusting for age, sex, BMI, total cholesterol, HDL-C, triglycerides and FBG.
[b]Logistic regression between mBMIΔ quintile and IFG adjusting for age, sex, BMI, total cholesterol, HDL-C, triglycerides and 2h-PLG. No adjustment for multiple comparisons was made.
CI = confidence interval
Significant p-values (two-sided) <0.05 are shown in bold.

Supplementary Table 8: The association between mBMIΔ and CVD (prevalent and incident) the AusDiab cohort

mBMIΔ	Prevalent CVD (n=577 cases versus 9690 controls)		10-year incident CVE (n=414 events versus 7936 non-events)		10-year incident IHD (n=304 events versus 8046 non-events)	
	**Odds ratio (95% CI)[a]**	**p-value**	**Hazard ratio (95% CI)[b]**	**p-value**	**Hazard ratio (95% CI)[c]**	**p-value**
mBMIΔ (SD)	1.3 (1.1, 1.4)	**1.28E-04**	1.0 (0.9, 1.2)	5.00E-01	1.1 (0.9, 1.2)	4.00E-01
Q1 (reference)	-	-	-	-	-	-
Q2	1.5 (1.0, 2.2)	5.80E-02	1.0 (0.7, 1.4)	9.00E-01	1.0 (0.7, 1.4)	8.00E-01
Q3	1.3 (0.9, 2.0)	1.51E-01	0.9 (0.6, 1.3)	5.00E-01	0.8 (0.6, 1.2)	3.00E-01
Q4	1.7 (1.1, 2.5)	**1.07E-02**	1.2 (0.9, 1.7)	2.00E-01	1.2 (0.8, 1.7)	3.00E-01
Q5	2.1 (1.5, 3.2)	**1.25E-04**	1.1 (0.8, 1.5)	6.00E-01	1.0 (0.7, 1.5)	9.00E-01

[a]Logistic regression between the quintiles of mBMIΔ and prevalent CVD adjusting for age, sex, BMI, smoking status, diabetes history plus clinical lipids (total cholesterol, HDL-C and triglycerides)
[b]Proportional hazard Cox-regression between the quintiles of mBMIΔ and major incident CVE adjusting for age, sex, BMI, smoking status and diabetes history plus clinical lipids (total cholesterol, HDL-C and triglycerides)
[c]Proportional hazard Cox-regression between the quintiles of mBMIΔ and incident IHD adjusting for age, sex, BMI, total cholesteroo, HDL-C, triglycerides, smoking status and diabetes history. No adjustment for multiple comparisons was made.
Significant p-values (two-sided) <0.05 are shown in bold.
CVD, Cardiovascular disease; CVE, Cardiovascular event; IHD, ischemic heart disease; Q, quintile

Supplementary Table 9: The association between mBMIΔ and CVD (prevalent and incident) the BHS cohort

mBMIΔ	Prevalent CVD (n=238 cases versus 4254 controls)		10-year incident CVE (IHD) (n=284 events versus 3970 non-events)	
	**Odds ratio (95% CI)[a]**	**p-value**	**Hazard ratio (95% CI)[c]**	**p-value**
mBMIΔ (SD)	1.19 (1.03, 1.37)	**2.1E-02**	1.2 (1.1, 1.3)	**5.00E-03**
Q1 (reference)	-	-	-	-
Q2	0.8 (0.5,1.4)	4.5E-01	1.2 (0.8, 1.9)	3.00E-01
Q3	1.6 (1.0, 2.6)	5.6E-02	1.8 (1.2, 2.7)	**9.00E-03**
Q4	1.4 (0.8, 2.2)	2.1E-01	1.8 (1.2, 2.8)	**4.00E-03**
Q5	1.4 (0.9, 2.3)	1.4E-01	1.7 (1.1, 2.6)	**1.10E-02**
	**Odds ratio (95% CI)[b]**	**p-value**	**Hazard ratio (95% CI)[d]**	**p-value**
mBMIΔ (SD)	1.1 (0.9, 1.3)	1.94E-01	1.1 (0.9, 1.2)	2.00E-01
Q1 (reference)	-	-	-	-
Q2	0.8 (0.5, 1.3)	3.57E-01	1.1 (0.7, 1.7)	7.00E-01
Q3	1.5 (0.9, 2.4)	9.70E-02	1.5 (1.0, 2.4)	**4.55E-02**
Q4	1.2 (0.8, 2.0)	4.36E-01	1.6 (1.0, 2.4)	**3.40E-02**
Q5	1.2 (0.7, 1.9)	5.57E-01	1.3 (0.8, 2.0)	3.00E-01

[a]Logistic regression between the quintiles of mBMIΔ and prevalent CVD adjusting for age, sex, BMI, smoking status and diabetes history.
[b]Logistic regression between the quintiles of mBMIΔ and prevalent CVD adjusting for age, sex, BMI, total cholesterol, HDL-C, triglycerides, smoking status and diabetes history.
[c] Proportional hazard Cox-regression between the quintiles of mBMIΔ and incident CVE adjusting for age, sex, BMI, smoking status and diabetes history.
[d]Proportional hazard Cox-regression between the quintiles of mBMIΔ and incident CVE adjusting for age, sex, BMI, total cholesteroo, HDL-C, triglycerides, smoking status and diabetes history. No adjustment for multiple comparisons was made.
Significant p-values (two-sided) <0.05 shown in bold.
CVD, Cardiovascular disease; CVE, Cardiovascular event; IHD, ischemic heart disease; Q, quintile

Supplementary Table 10: The association of BMI, mBMI and mBMIΔ with disease outcomes in the AusDiab participants

Outcome	BMI[a]		mBMI[b]		mBMIΔ[c]	
	**Odds ratio (95% CI)**	**p-value**	**Odds ratio (95% CI)**	**p-value**	**Odds ratio (95% CI)**	**p-value**
Newly diagnosed prevalent T2DM (n=395 cases versus 7,733 NGT)	2.36 (2.12–2.63)	**1.38E-55**	2.65 (2.36–2.97)	**5.11E-62**	1.69 (1.51–1.88)	**6.48E-21**
5-year incident T2DM (n=218 cases versus 5354 controls)	1.83 (1.60–2.08)	**2.46E-19**	1.95 (1.70–2.24)	**1.62E-21**	1.34 (1.17–1.54)	**2.34E-05**
Prevalent CVD (n=577 cases versus 9690 controls)[d]	1.29 (1.13–1.47)	**2.59E-04**	1.4 (1.23–1.60)	**1.74E-09**	1.29 (1.14–1.44)	**3.40E-05**
	Hazard ratio (95% CI)		Hazard ratio (95% CI)		Hazard ratio (95% CI)	
10 year incident CVE (n=414 events versus 7936 non-events)[d]	1.13 (1.01–1.27)	**2.70E-02**	1.36 (0.99–1.85)	5.45E-02	1.11 (1.0–1.22)	**4.30E-02**

[a] Logistic/Cox regression between cardiometabolic outcomes and BMI adjusted for age, sex and mBMIΔ.

[b]Logistic/Cox regression between cardiometabolic outcomes and mBMI adjusted for age, and sex.

[c]Logistic/Cox regression between cardiometabolic outcomes and mBMIΔ adjusted for age, sex, and BMI

[d]The model includes diabetes history and smoking as covariates in addition to the covariates in the models a, b and c. No adjustment for multiple comparisons was made.

Significant p-values (two-sided) <0.05 are shown in bold.

Supplementary Table 11: The effect of mBMIΔ on logistic models to predict prevalent and incident T2DM and CVD

Model[a]	Associations with newly diagnosed prevalent T2DM (n=395 /7733 NGT)			Likelihood ratio test (LRT)	Associations with 5-year incident T2DM (n=218/5354 controls)			Likelihood ratio test (LRT)	Associations with prevalent CVD (n=577/9690)			Likelihood ratio test (LRT)
	**Odds ratio (95% CI)**	**p-value**	**AIC**	**p-value**	**Odds ratio (95% CI)**	**p-value**	**AIC**	**p-value**	**Odds ratio (95% CI)**	**p-value**	**AIC**	**p-value**
Age	2.7 (2.4, 3.0)	**5.96E-64**	2652.4	**8.02E-13**	1.6 (1.3, 1.8)	**2.33E-08**	1742.4	**7.98E-04**	3.38 (3.03, 3.77)	**4.56E-106**	3269.9	**6.02E-05**
Sex (Male)	1.5 (1.2, 1.8)	**6.76E-04**			1.3 (1.0, 1.76)	**4.82E-02**			1.94 (1.61, 2.34)	**6.15E-12**		
BMI	2.1 (1.9, 2.3)	**2.66E-49**			1.7 (1.5, 2.0)	**6.41E-19**			1.23 (1.11, 1.35)	**3.52E-05**		
**Model[b]**												
Age	2.7 (2.4, 3.0)	**9.65E-63**	2603.1		1.6 (1.3, 1.8)	**4.88E-08**	1733.1		3.41 (3.06, 3.82	**1.34E-105**	3254.8	
Sex (Male)	1.6 (1.3, 2.0)	**5.06E-05**			1.4 (1.0, 1.8)	**2.98E-02**			1.96 (1.62, 2.37)	**3.24E-12**		
BMI	2.2 (2.0, 2.5)	**7.69E-52**			1.8 (1.6, 2.0)	**3.46E-19**			1.27 (1.15, 1.40)	**3.62E-06**		
mBMIΔ	2.4 (1.9, 3.0)	**1.07E-12**			1.7 (1.2, 2.3)	**7.53E-04**			1.22 (1.11, 1.34)	**3.94E-05**		

[a]Logistic regression between outcomes and metabolic risk factors adjusted for age and sex and BMI

[b]Logistic regression between outcomes and metabolic risk factors adjusted for age and sex, BMI and mBMIΔ

T2DM, Type 2 diabetes mellitus; NGT, Normal glucose tolerant; CVD, Cardiovascular disease; AIC, Akaike's information criteria

Significant p-values (two-sided) <0.05 are shown in bold.

Supplementary Table 12: The association between anthropometric measures, diet and lifestyle factors and mBMIΔ.

Variable	Beta coefficient (95% CI)[a]	p-value
Age	-0.015 (-0.079, 0.048)	6.39E-01
Sex	0.253 (0.136, 0.371)	**2.29E-05**
BMI	-0.038 (-0.048, -0.027)	**2.65E-12**
Current smoker	-0.195 (-0.34, -0.051)	**8.14E-03**
PA time (Q2 relative to Q1)	0.068 (-0.087, 0.223)	3.90E-01
PA time (Q3 relative to Q1)	-0.113 (-0.267, 0.042)	1.53E-01
PA time (Q4 relative to Q1)	-0.244 (-0.4, -0.089)	**2.10E-03**
PA time (Q5 relative to Q1)	-0.51 (-0.67, -0.35)	**4.39E-10**
TV time (Q2 relative to Q1)	0.202 (0.07, 0.335)	**2.81E-03**
TV time (Q3 relative to Q1)	0.298 (0.158, 0.439)	**3.05E-05**
TV time (Q4 relative to Q1)	0.352 (0.177, 0.526)	**8.26E-05**
TV time (Q5 relative to Q1)	0.514 (0.311, 0.716)	**6.79E-07**
SBP	0.194 (0.133, 0.254)	**3.60E-10**
Family history of diabetes	0.237 (0.108, 0.365)	**3.04E-04**
History of CVD	0.271 (0.051, 0.491)	**1.56E-02**
Alcohol (100g/d)	0.045 (-1.147, 1.237)	9.40E-01
Level of education (Never to some high school, completed university or equivalent)	-0.073 (-0.175, 0.029)	1.60E-01
Red meat (100g/d)	0.102 (0.001, 0.203)	**4.86E-02**
Processed meat (100g/d)	0.345 (0.067, 0.623)	**1.50E-02**
Fish (g/d)	-0.001 (-0.004, 0.001)	2.45E-01
All Fat (100g/d)	-0.503 (-1.069, 0.062)	8.10E-02
Fibre intake (Q2 relative to Q1)	-0.154 (-0.321, 0.012)	6.98E-02
Fibre intake (Q3 relative to Q1)	-0.288 (-0.482, -0.094)	**3.66E-03**
Fibre intake (Q4 relative to Q1)	-0.484 (-0.712, -0.257)	**2.99E-05**
Fibre intake (Q5 relative to Q1)	-0.622 (-0.912, -0.333)	**2.54E-05**
Carbohydrate (g/d)	0.057 (-0.023, 0.138)	1.64E-01
Sugars (g/d)	-0.038 (-0.123, 0.046)	3.75E-01
Protein (g/d)	-0.504 (-1.111, 0.102)	1.03E-01
Energy intake (100g/d)	0.008 (0.001, 0.015)	**3.62E-02**
Total vegetable intake (100g/d)	0.085 (-0.014, 0.184)	9.19E-02
Total fruit intake (Q2 relative to Q1)	-0.052 (-0.209, 0.105)	5.14E-01
Total fruit intake (Q3 relative to Q1)	-0.128 (-0.292, 0.036)	1.27E-01
Total fruit intake (Q4 relative to Q1)	-0.25 (-0.423, -0.076)	**4.76E-03**
Total fruit intake (Q5 relative to Q1)	-0.25 (-0.443, -0.057)	**3.90E-03**

[a]Multiple linear regression of mBMIΔ against anthropometric measures, diet and lifestyle factors.
Significant p-values (two-sided) <0.05 are shown in bold. No adjustment for multiple comparisons was made.
CI-confidence interval; PA, physical activity; TV time, television viewing time

Supplementary Figures

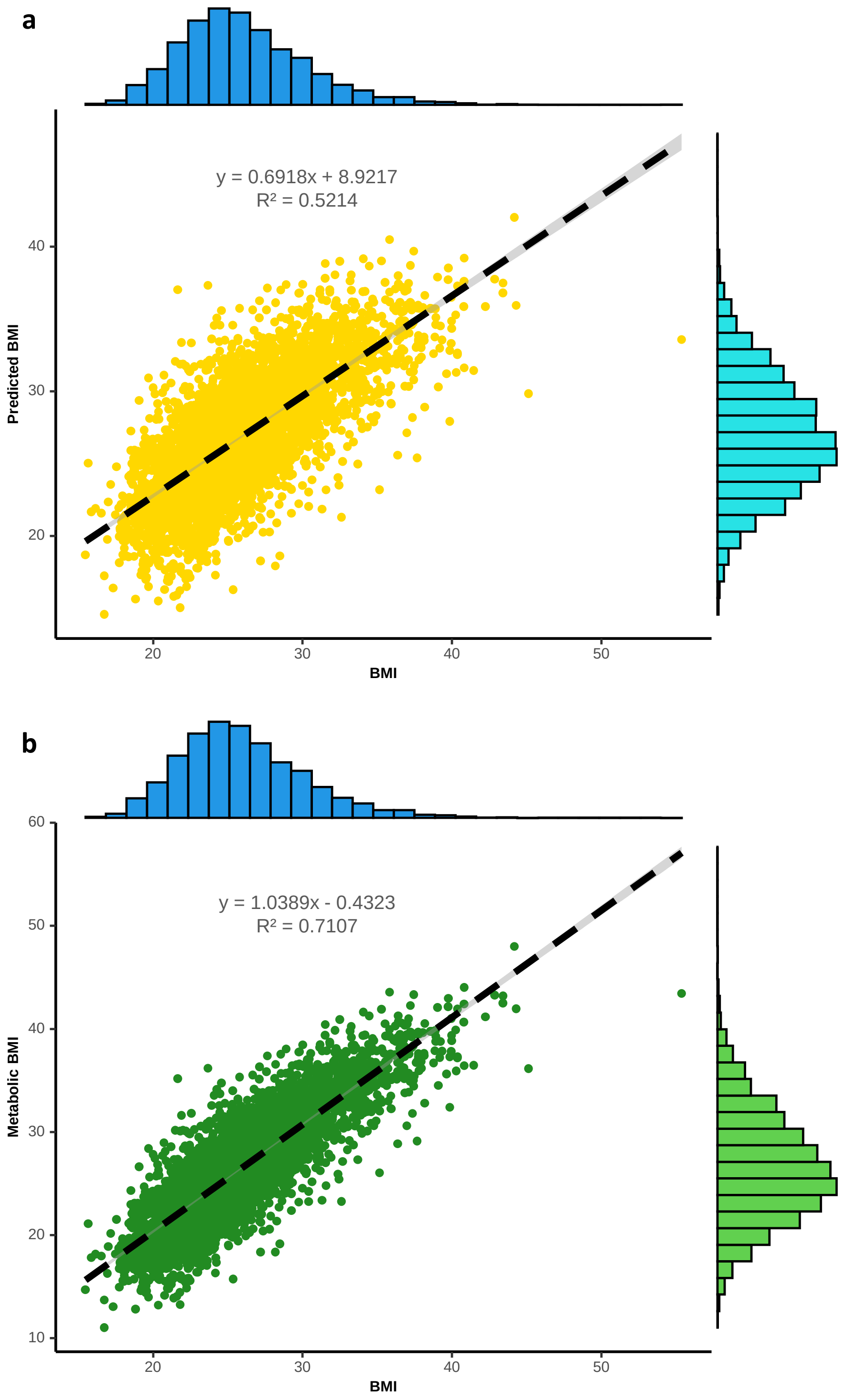

Supplementary Fig.1 Validation of the ridge model for the prediction of BMI in the BHS cohort (n=4,492 independent subjects). a The correlation between true BMI (x-axis) and predicted BMI using lipidomic data (y-axis). **b** The correlation between true BMI (x-axis) and metabolic BMI (y-axis)

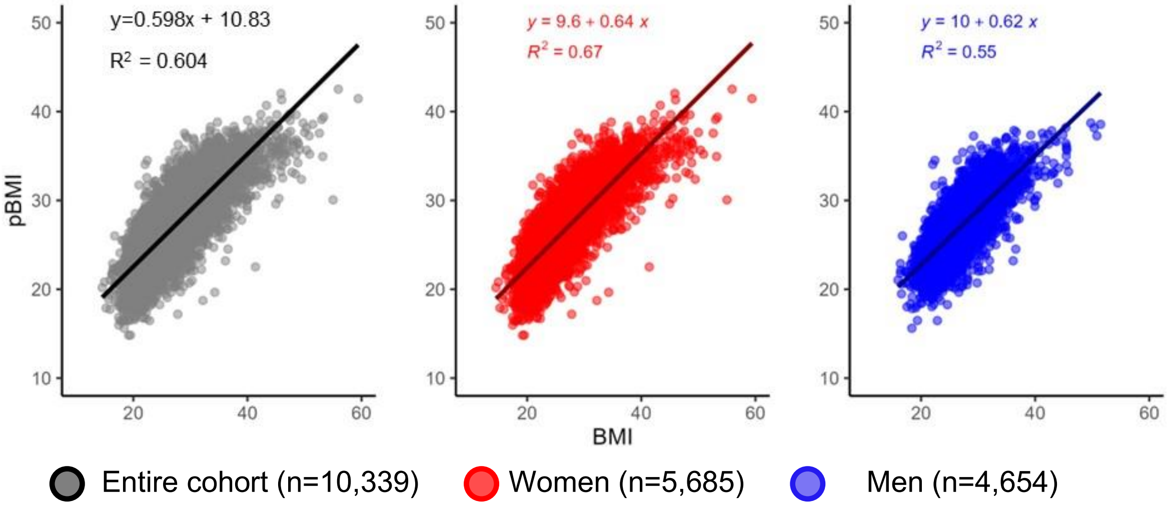

Supplementary Fig.2 Ridge prediction of BMI using lipidomic data in the AusDiab cohort (n=10,339 independent subjects). The correlation (R^2) between true BMI (x-axis) and predicted BMI (y-axis) in the entire cohort (left panel), in women (middle panel) and in men (right panel).

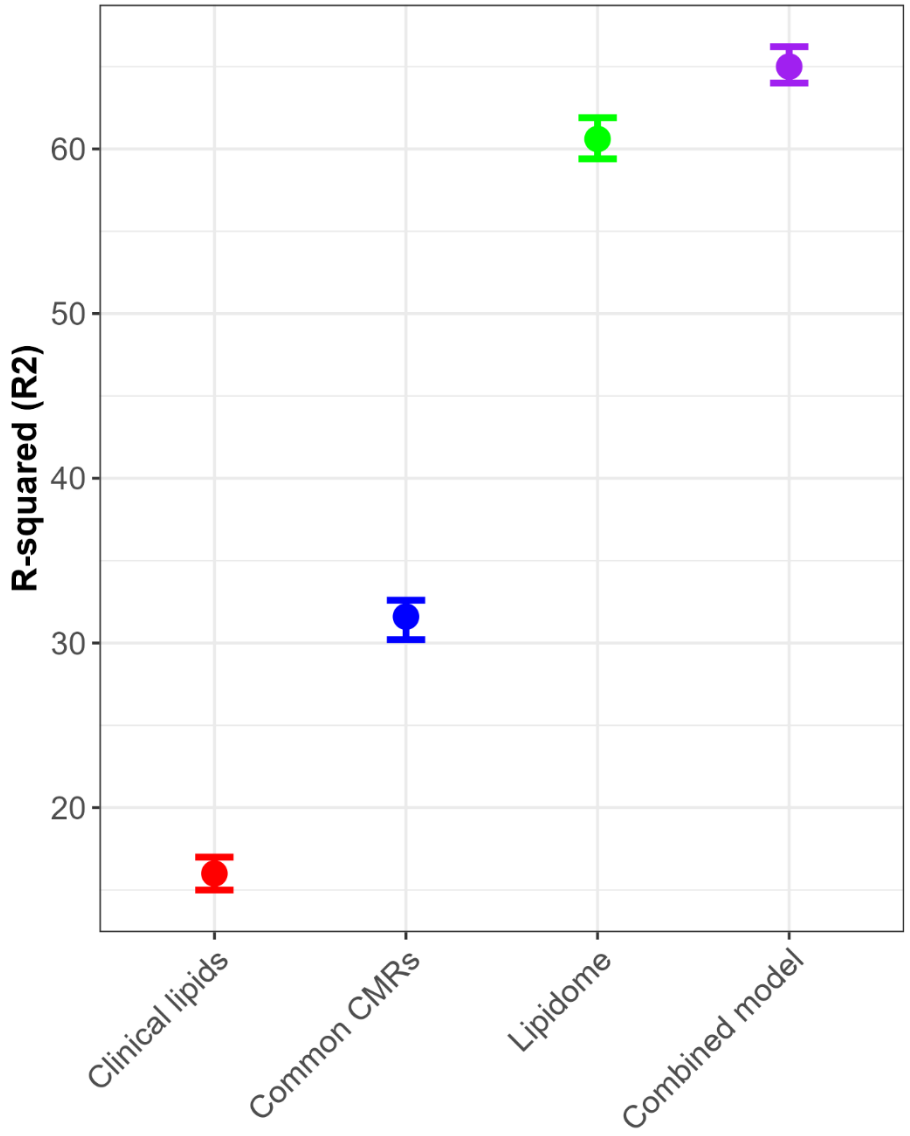

Supplementary Fig. 3 BMI modelling: Prediction performance BMI model using clinical lipids, common CMRs, lipidomics and all- combined using ridge model in the AusDiab cohort (n=10,339 independent subjects). Ridge regression was used to predict BMI. The red, blue, green and purple circles represent the R^2 of explained variation of BMI using clinical lipids, CMRs, the lipidome and combination of these respectively. The error bars represent 95% confidence intervals for R^2. Clinical lipid refers to total cholesterol, HDL-C and triglycerides. CMRs (cardiometabolic risk factors) including age, sex, total cholesterol, HDL-C, triglycerides, fasting blood glucose, 2h-post load glucose, glycated haemoglobin 1, HOMA-IR, systolic blood pressure and diastolic blood pressure. Combined model is the model with CMRs and the lipidome.

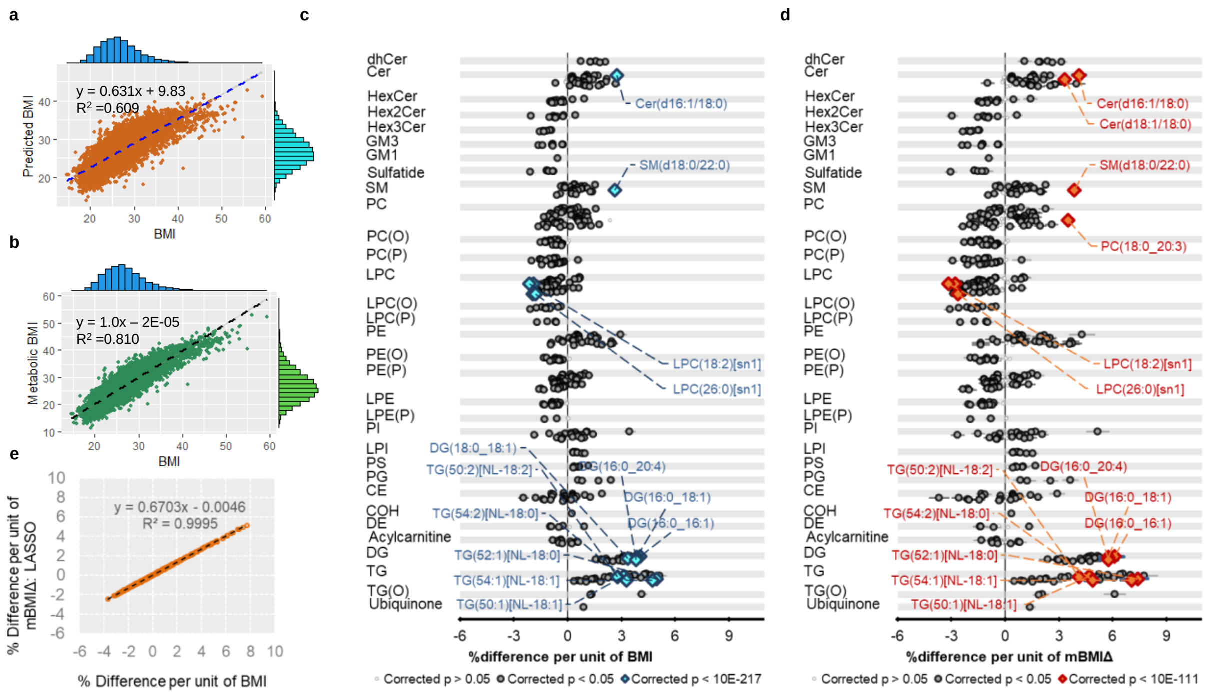

Supplementary Fig.4 Modelling of the metabolic BMI score and comparison of the captured lipid biology with BMI using LASSO. a Correlation between measured BMI and predicted BMI in the AusDiab cohort (n = 10,339 independent subjects). **b** Correlation between measured BMI and metabolic BMI (mBMI) in the AusDiab cohort (n = 10,339). **c** Associations of BMI with plasma lipid species and (**d**) Association of mBMIΔ with plasma lipid species using linear regression analysis adjusting for age and sex. Grey open circles show species ($p>0.05$), grey and dark closed circles show species with $p<0.05$ after correction for multiple comparisons using the method of Benjamini and Hochberg. Blue circles and brown diamonds represent the top 15 most significant lipids associated with BMI ($p<$10E-217) and mBMIΔ ($p<$10-111) respectively. The whiskers represent 95% confidence intervals. **e** The correlation between effect sizes of each lipid associated with BMI, x-axis and with mBMIΔ, y- axis. AC = acylcarnitine, CE = cholesteryl ester, Cer = ceramide, COH = cholesterol, DE = dehydrocholesterol, dhCer = dihydroceramide, DG = diacylglycerol, GM1 = GM1 ganglioside, GM3 = GM3 ganglioside, HexCer = monohexosylceramide, Hex2Cer = dihexosylceramide, Hex3Cer = trihexosylceramide, LPC = lysophosphatidylcholine, LPC(O) = lysoalkylphosphatidylcholine, LPC(P) = lysoalkenylphosphatidylcholine, LPE = lysophosphatidylethanolamine, LPE(P) = lysoalkenylphosphatidylethanolamine, LPI = lysophosphatidylinositol, PC = phosphatidylcholine, PC(O) = alkylphosphatidylcholine, PC(P) = alkenylphosphatidylcholine, PE = phosphatidylethanolamine, PE(O) = alkylphosphatidylethanolamine, PE(P) = alkenylphosphatidylethanolamine, PG = phosphatidylglycerol, PI = phosphatidylinositol, PS = phosphatidylserine, SHexCer = sulfatide, SM = sphingomyelin, TG = triacylglycerol, TG(O) = alkyl-diacylglycerol.

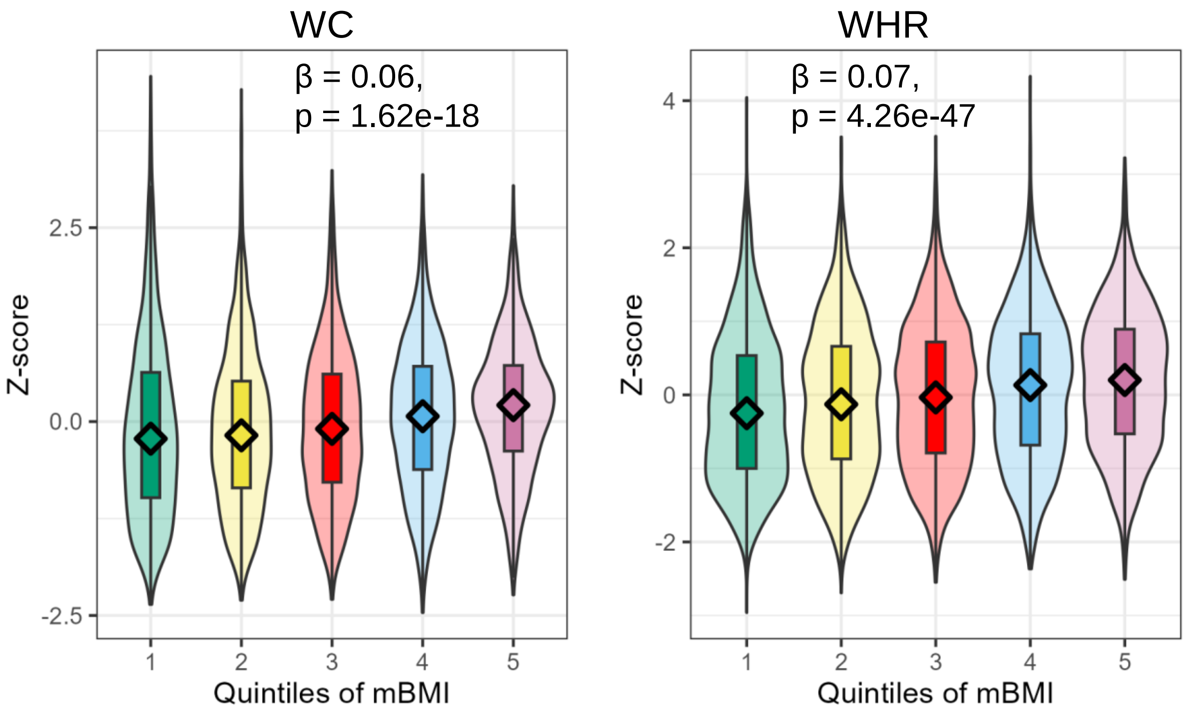

Supplementary Fig.5 The relationship of mBMIΔ with WC and WHR in the AusDiab (n=10,339 independent subjects). Violin and box plots represent the distribution of z-scores of WC (left) and WHR (right). The data depicted in the box and whisker plots span from the minimum to the maximum values (z-score). The lower and upper boundaries of the box correspond to the 25th and 75th percentiles, respectively, and the central open diamonds within the boxes represent the median values. Linear regression analyses of mBMIΔ quintile (predictor) against WC/WHR (outcome) were performed. β-coefficients and p-values from the linear regression analyses are presented. WC, waist circumference, WHR, waist-to-hip ratio.

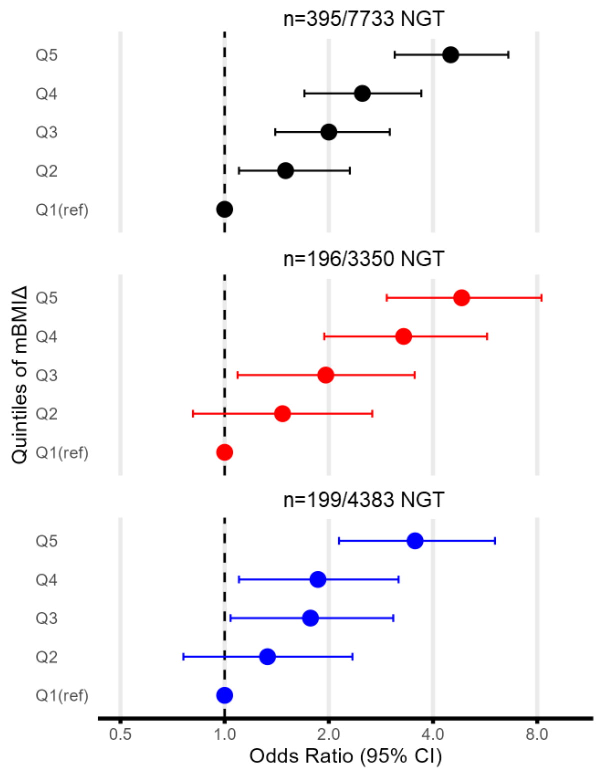

mBMIΔ quintile	OR (95% CI)	p-value
Q5	4.5 (3.1, 6.6)	1.48E-15
Q4	2.5 (1.7, 3.7)	3.65E-06
Q3	2.0 (1.4, 3.0)	6.42E-04
Q2	1.5 (1.01, 2.3)	4.42E-02
Q1 (ref)	1	-

	OR (95% CI)	p-value
Q5	4.8 (2.9, 8.2)	1.66E-09
Q4	3.3 (1.9, 5.7)	1.59E-05
Q3	2.0 (1.1, 3.5)	2.48E-02
Q2	1.5 (0.8, 2.7)	2.04E-01
Q1 (ref)	1	-

	OR (95% CI)	p-value
Q5	3.6 (2.1, 6.0)	1.56E-06
Q4	1.9 (1.1, 3.2)	2.20E-02
Q3	1.8 (1.04, 3.1)	3.81E-02
Q2	1.3 (0.8, 2.3)	3.14E-01
Q1 (ref)	1	-

Supplementary Fig.6 The relationship between mBMIΔ and T2DM. A logistic regression between prevalent diabetes and controls was performed adjusted for age, sex, and BMI (entire cohort, n=10,399 independent subjects) and age and BMI (for sex specific analyses). The odds ratios associated with moving from the reference quintile (Q1) to Q2 to Q5 (x-axis) and the quintiles of mBMIΔ (y-axis) for the entire cohort (top panel), women (middle panel) and men (bottom panel). Error bars represent the 95% CIs.

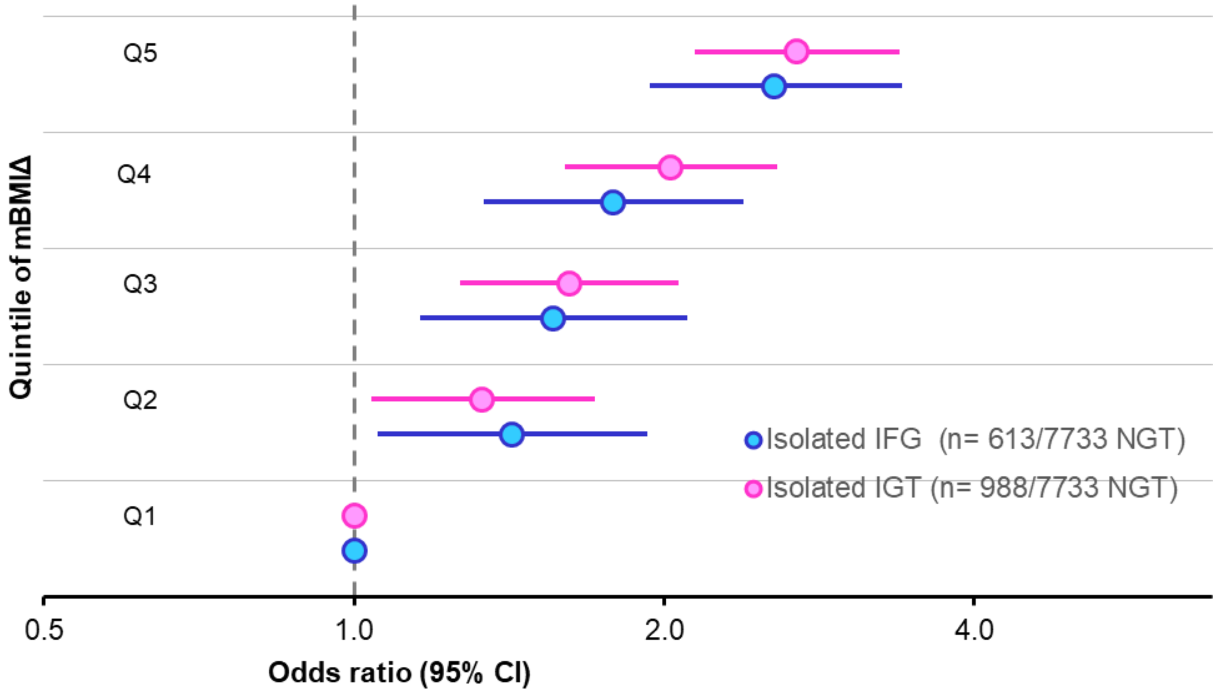

Supplementary Fig.7 The relationship between mBMIΔ and IGT or IFG. Depicted on the x-axis are the odds ratios and error bars (95% CIs) for the isolated IGT (pink circles) and IFG (sky-blue circles) across the quintiles of mBMIΔ (y- axis). The odds ratios were computed using a logistic regression between isolated IGT, n = 988/7, 733 NGT or IFG, n = 613/7,733 NGT independent subjects and quintile of the mBMIΔ adjusting for age, sex, and BMI.

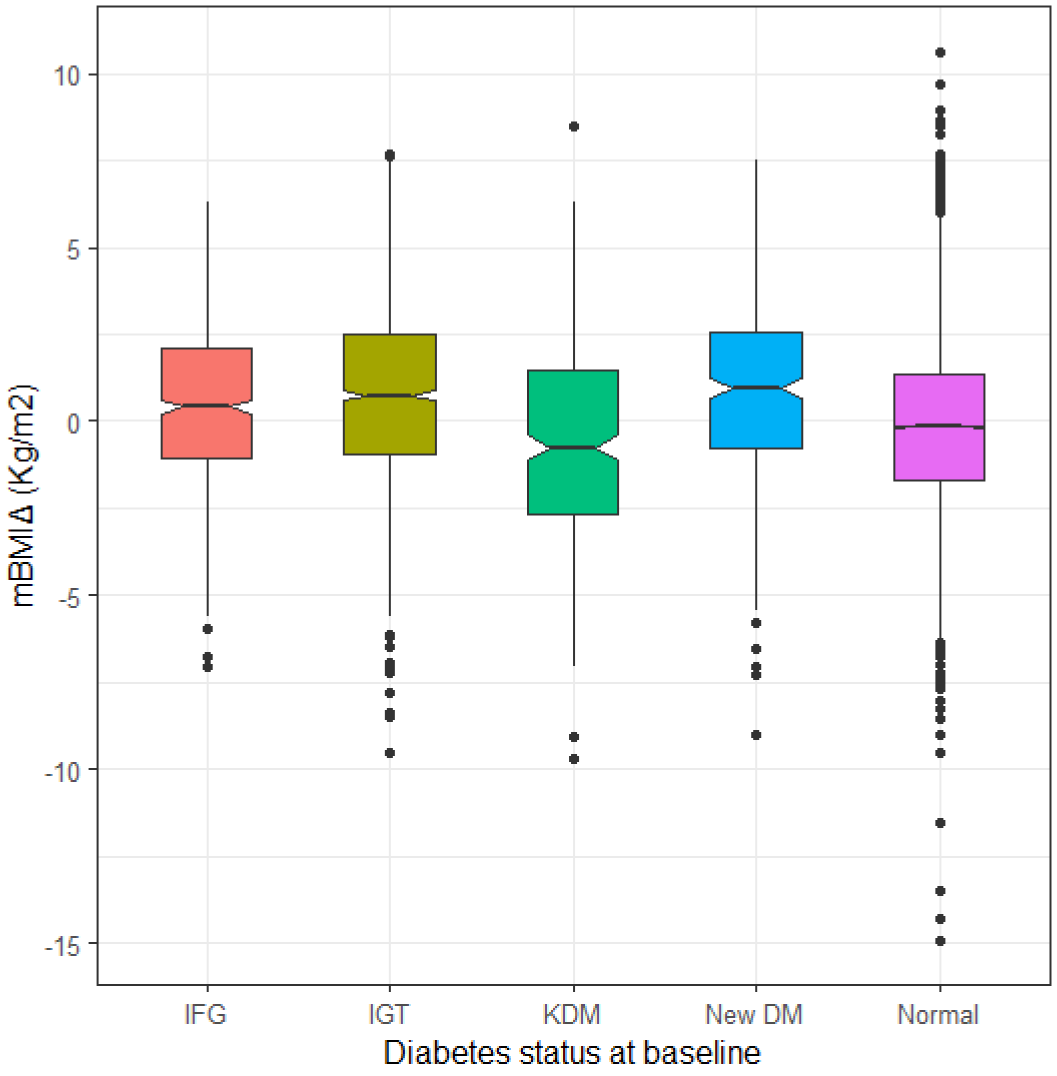

Supplementary Fig.8 The relationship between mBMIΔ and type 2 diabetes status at baseline in the AusDiab cohort (n=10,339 independent subjects). Box plots represent the distribution of mBMIΔ (y-axis) stratified by diabetes status (x-axis). The box and error bars span from the minimum to the maximum values (kg/m2 of mBMI**Δ**). The lower and upper boundaries of the box correspond to the 25th and 75th percentiles, respectively, and the central line within the boxes represent the median values. Abbreviations: IFG, impaired fasting glucose; IGT, impaired glucose tolerant; KDM, known diabetes mellitus; New DM, new diabetes mellitus.

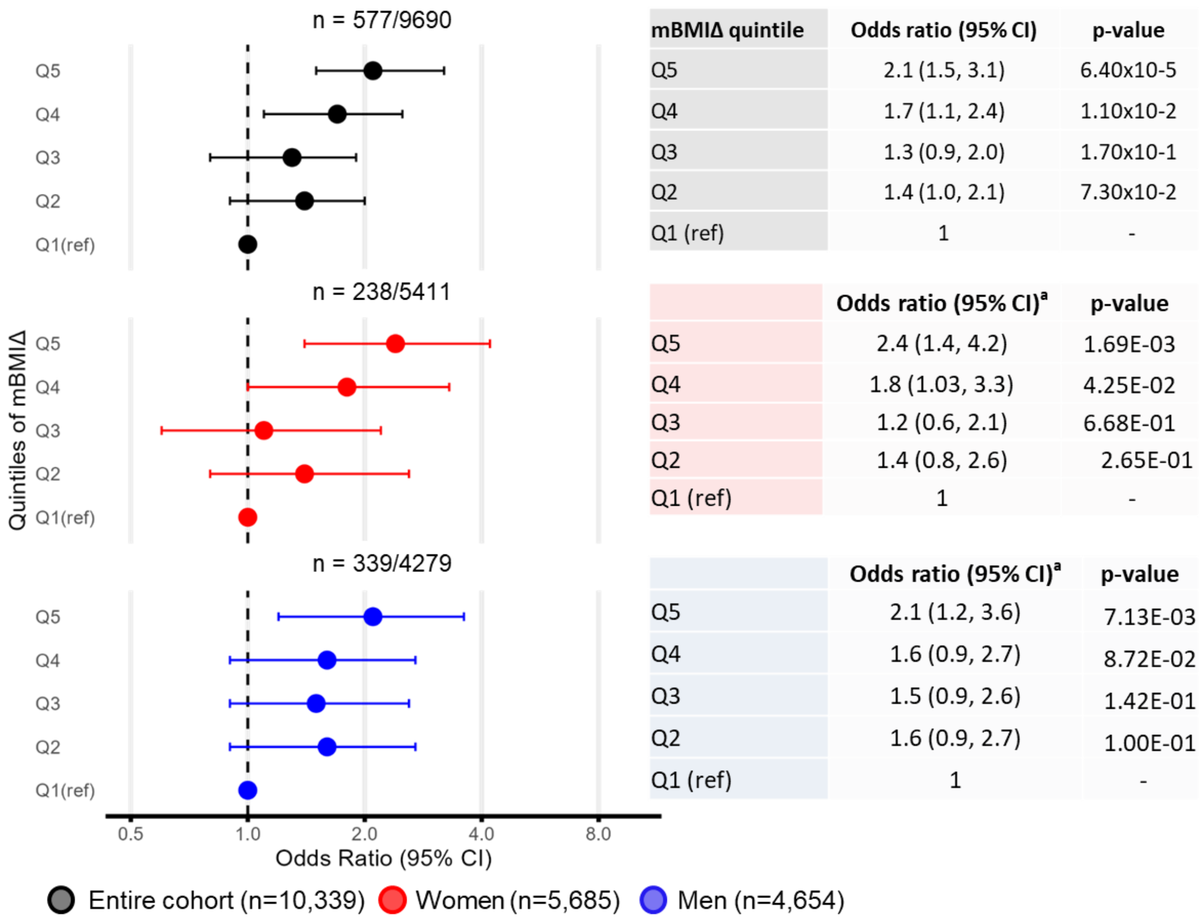

mBMIΔ quintile	Odds ratio (95% CI)	p-value
Q5	2.1 (1.5, 3.1)	6.40x10-5
Q4	1.7 (1.1, 2.4)	1.10x10-2
Q3	1.3 (0.9, 2.0)	1.70x10-1
Q2	1.4 (1.0, 2.1)	7.30x10-2
Q1 (ref)	1	-

	Odds ratio (95% CI)[a]	p-value
Q5	2.4 (1.4, 4.2)	1.69E-03
Q4	1.8 (1.03, 3.3)	4.25E-02
Q3	1.2 (0.6, 2.1)	6.68E-01
Q2	1.4 (0.8, 2.6)	2.65E-01
Q1 (ref)	1	-

	Odds ratio (95% CI)[a]	p-value
Q5	2.1 (1.2, 3.6)	7.13E-03
Q4	1.6 (0.9, 2.7)	8.72E-02
Q3	1.5 (0.9, 2.6)	1.42E-01
Q2	1.6 (0.9, 2.7)	1.00E-01
Q1 (ref)	1	-

Supplementary Fig. 9 The relationship between mBMIΔ and prevalent CVD. A logistic regression was performed between CVD and controls for the entire cohort (top panel, n =10,339 independent samples), women (middle panel) and men (bottom panel) djusted for age, sex, BMI, smoking and diabetes. Each circle and the associated error bars represent the odds ratio and 95% CI (x-axis) for the prevalent CVD across the quintiles of mBMIΔ (y-axis).

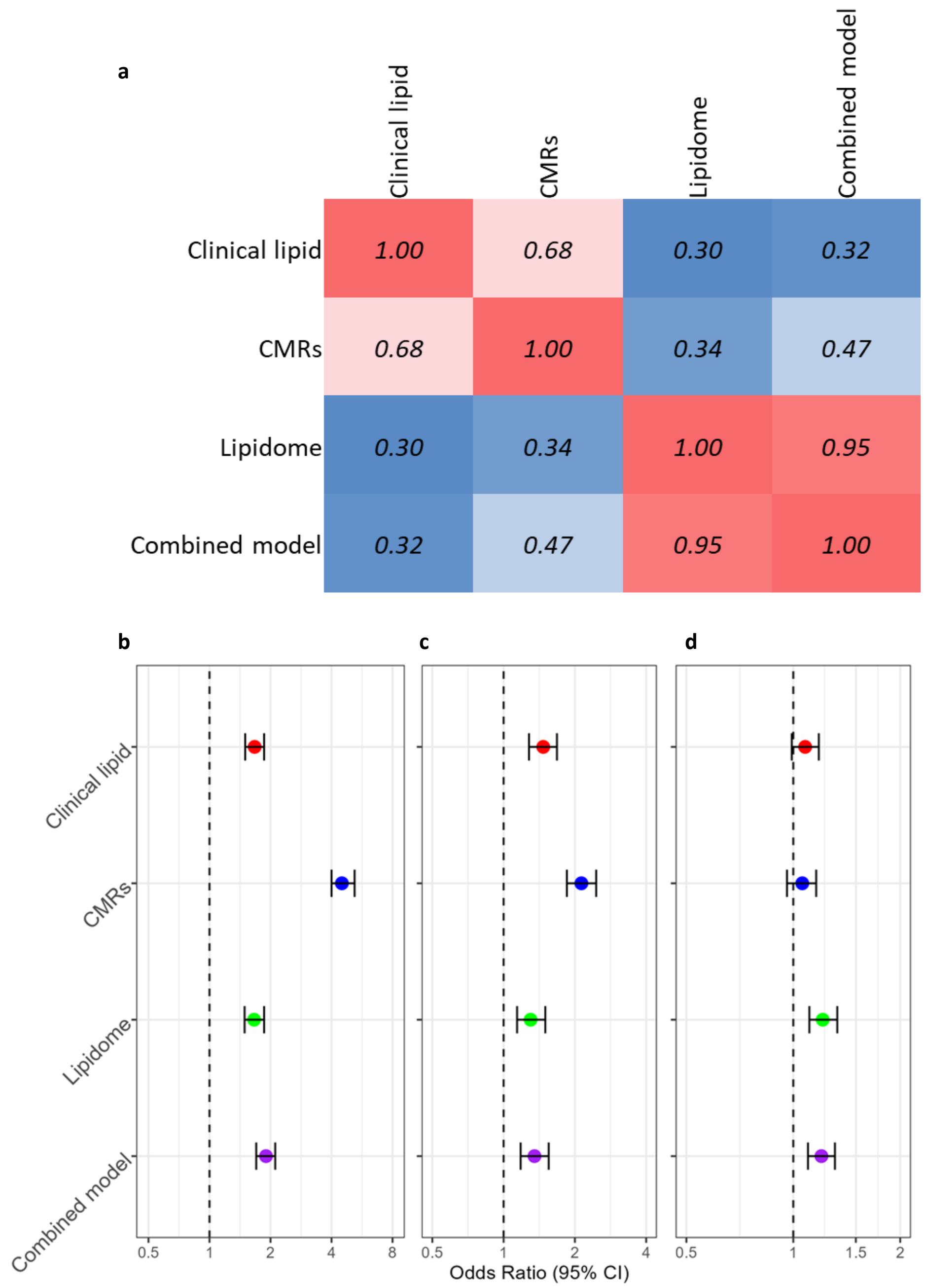

Supplementary Fig.10 mBMIΔ calculated using different sets of predictors and the relationship with disease outcomes in the AusDiab cohort (n=10,399 independent samples). a Pearsons's correlation coefficients (R) among the mBMIΔs calculated using different sets of predictors; clinical lipids, CMRs, lipidome and combined model**.** (**b, c**) Age, sex and BMI adjusted associations of mBMIΔ with prevalent T2DM and incident T2DM respectively. **d** age, sex, BMI, diabetes and smoking adjusted prevalent CVD. Each circle represents odds ratio and the error bars represent 95% CIs for mBMIΔ derived using the clinical lipids (red), CMRs (blue), lipidome (green) and combined model (purple). The odds ratios are computed using a logistic regression analysis. Clinical lipid refers to total cholesterol, HDL-C and triglycerides. CMRs (cardiometabolic risk factors) including age, sex, total cholesterol, HDL-C, triglycerides, fasting blood glucose, 2h-post load glucose, glycated haemoglobin 1, HOMA-IR, systolic blood pressure and diastolic blood pressure. Combined model is the model with CMRs and the lipidome

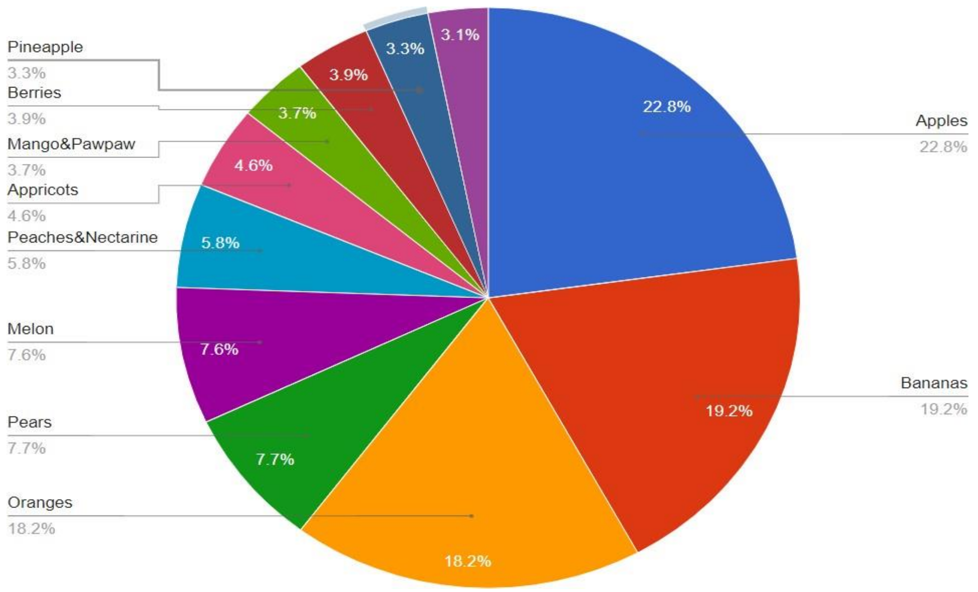

Supplementary Fig.11 Percentage contribution of individual fruits to the total fruit intake in the AusDiab cohort (n=10,339 independent samples).

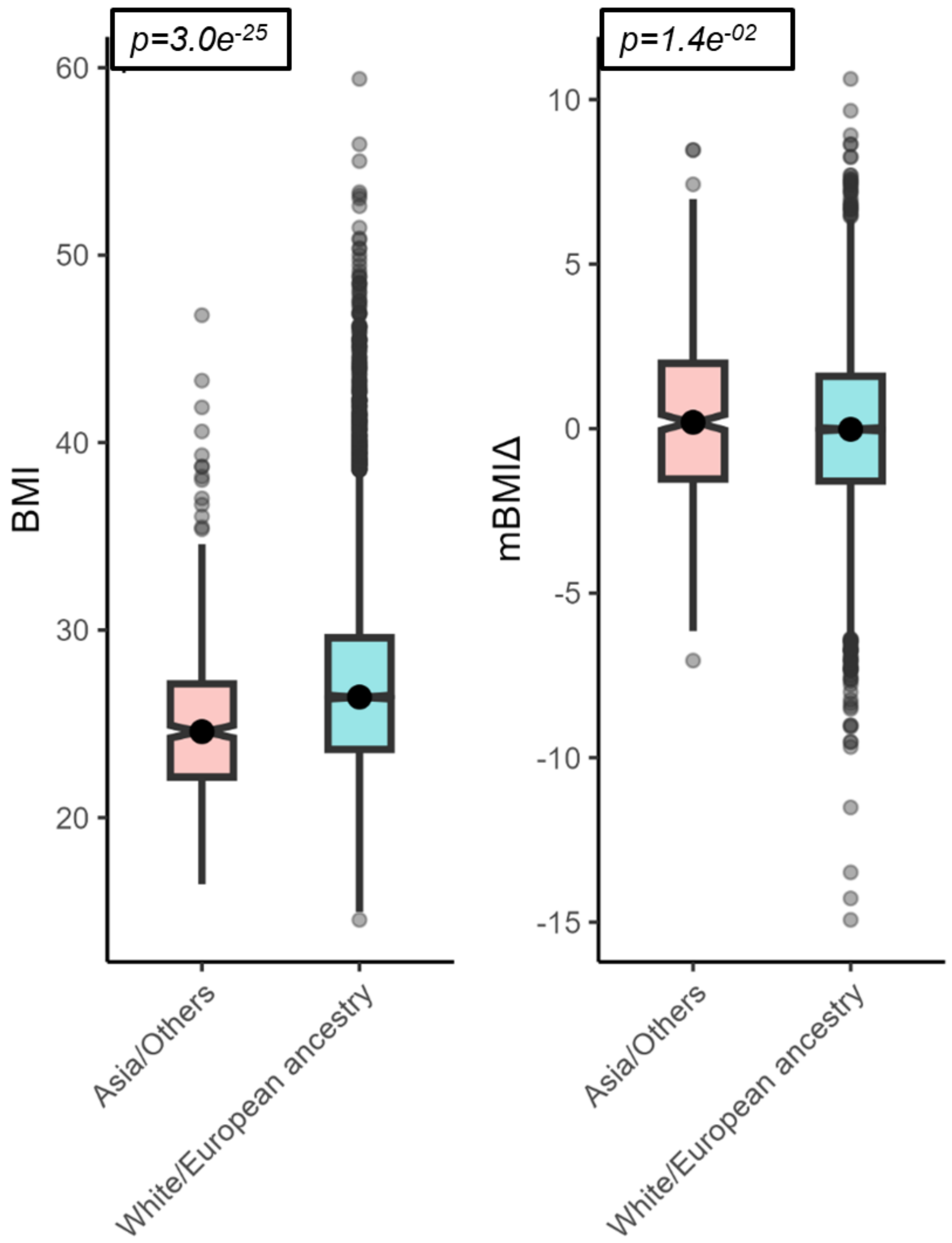

Supplementary Fig.12 Differences in BMI and mBMIΔ between ethnicities in the AusDiab cohort (n=10,339 independent samples). White/European ancestry (n=9786) (94.7%) and Asian/other (n = 553) (5.3%). The box plots and error span from the minimum to the maximum values (BMI, kg/m2) (left panel) and mBMIΔ (right panel). The lower and upper boundaries of the box correspond to the 25th and 75th percentiles, respectively, and the central black circles within the boxes represent the median values. Two-sided p-values from an independent students T-test are displayed on the top left of each figure.

Supplementary Note 1: Derivation of metabolic BMI delta (mBMIΔ)

Let y be the vector of BMI measurements $(n \times 1)$, X is a matrix of all predictors (intercept + lipid species + covariates; $n \times p + 1$). We create a regularized linear model of BMI to estimate the predictor beta coefficients:

$$\hat{\beta} = (X^t X + \lambda I)^{-1} X^t y$$

This is equivalent to minimizing the equation:

$$\min_{\hat{\beta}} \mathbb{E} \left(y - X\hat{\beta}\right)^2 + \lambda \hat{\beta}^T \hat{\beta}$$

Using these estimated beta coefficients, the predicted BMI is calculated:

$$\hat{y} = X(X^t X + \lambda I)^{-1} X^t y$$

$$= X\hat{\beta}$$

We then proceed to estimate predicted BMI ($\hat{y}$) using actual BMI measurements, using the model:

$$\hat{y} = \mu^* + y\beta^* + \varepsilon$$

Where β^* is a scaling factor and u^* is the intercept. The mBMIΔ (residual) can be expressed as:

$$r = \hat{y} - (\mu^* + y\beta^*)$$

Estimation of predicted BMI involves minimizing the equation:

$$\min_{\beta^*, u^*} \mathbb{E} \left(\hat{y} - (u^* + y\beta^*)\right)^2$$

Substituting predicted BMI for our original model:

$$\min_{\beta^*, u^*} \mathbb{E} \left(X\hat{\beta} - (u^* + y\beta^*)\right)^2$$

$$= \min_{\beta^*, u^*} \mathbb{E} \left(X \begin{bmatrix} \hat{\beta}_u - u^* \\ \hat{\beta}_1 \\ \vdots \\ \hat{\beta}_p \end{bmatrix} - y\beta^* \right)^2$$

$$= \min_{\beta^*, u^*} \mathbb{E}\, \beta^{*2} \left(X \begin{bmatrix} (\hat{\beta}_u - u^*)/\beta^* \\ \hat{\beta}_1/\beta^* \\ \vdots \\ \hat{\beta}_p/\beta^* \end{bmatrix} - y \right)^2$$

We can see the least squares solution involves scaling the predicted BMI beta coefficients. This explains why the plot of the beta coefficients (Figure 2E) produces a straight line. The larger coefficients observed with mBMIΔ compared to BMI result from the fact that the variance in the mBMIΔ can be fully explained by the lipid species whereas only ~60% of the variance in BMI is explained by the lipid species.

Supplementary Note 2: Dietary and lifestyle assessment

Dietary intake was assessed using a standard, semi-quantitative, self-administered food frequency questionnaire (FFQ) developed by the Cancer Council of Victoria [1-3]. This questionnaire had 74 food items with 10 frequency response options ranging from "never" to "three or more times per day" and was originally developed to estimate macro- and micronutrient intake in adult Australians from diverse backgrounds [1]. Briefly, participants were asked to mention their usual frequency of intake of food items, in the last 12 months. Daily nutrient and energy intake was computed by multiplying the frequency of food by the standard portion size for each of the food items followed by conversion of the nutrient intake to (g/day) based on the NUTTAB95 nutrient composition database [4].

"Active Australia questionnaire" was used to collect physical activity time. Participants were asked about their engagements in leisure-time physical activities (including walking for transport) during the previous week [5]. Time spent walking or performing moderate-intensity physical activity, plus double the time spent in vigorous intensity physical activity were summed up to obtain total physical activity time [6]. We created five categories of activity levels (<0.1, 0.1 to <0.3, 0.3 to <0.6, 0.6 to <1.12 and ≥ 1.12 hrs/day) based on the previously identified associations with cardio-metabolic risk factors [7].

Total time spent watching television or videos in the past 7 days was also collected using a previously validated instrument [8,9]. The total television viewing times were converted to hours/day. We created five categories of television viewing time (<1.0, 1.0 to <2, 2.0 to <3, 3.0 to <4.0, and ≥4 hrs/day).

Supplementary References

1. Ireland, P. et al. Development of the Melbourne FFQ: a food frequency questionnaire for use in an Australian prospective study involving an ethnically diverse cohort. Asia Pac J Clin Nutr **3**, 19-31 (1994).
2. Woods, R.K. et al. A valid food frequency questionnaire for measuring dietary fish intake. Asia Pac J Clin Nutr **11**, 56-61 (2002).
3. Tandon, N. et al. Effects of a Lifestyle Intervention to Prevent Deterioration in Glycemic Status Among South Asian Women With Recent Gestational Diabetes: A Randomized Clinical Trial. JAMA Network Open **5**, e220773-e220773 (2022).
4. Lewis J, Milligan G, Hunt A: NUTTAB95 Nutrient Data Table for Use in Australia. Canberra, Australia, Australian Government Publishing Service, 1995.
5. Australian Institute of Health and Welfare: The Active Australia Survey: A Guide and Manual for Implementation, Analysis and Reporting. Canberra, Australia, Australia Institute of Health and Welfare, 2003.
6. Armstrong T, Bauman A, Davies J: Physical Activity Patterns of Australian Adults: Results of the 1999 National Physical Activity Survey. Canberra, Australia, Australian Institute of Health and Welfare, 2000 (AIHW cat. no. CVD 10).
7. Healy, G.N., Dunstan, D.W., Shaw, J.E., Zimmet, P.Z. & Owen, N. Beneficial Associations of Physical Activity With 2-h but Not Fasting Blood Glucose in Australian Adults: The AusDiab Study. Diabetes Care **29**, 2598-2604 (2006).
8. Salmon, J., Owen, N., Crawford, D., Bauman, A. & Sallis, J.F. Physical activity and sedentary behavior: a population-based study of barriers, enjoyment, and preference. Health Psychol **22**, 178-188 (2003).
9. Margeirsdottir, H.D., Larsen, J.R., Brunborg, C., Sandvik, L. & Dahl-Jørgensen, K. Strong association between time watching television and blood glucose control in children and adolescents with type 1 diabetes. Diabetes Care **30**, 1567-1570 (2007).
