# Supplementary material for: Metabolic phenotyping of BMI to characterize cardiometabolic risk: evidence from large population-based cohorts
Source: Nat Commun. 2023 Oct 7;14:6280. doi: 10.1038/s41467-023-41963-7 (PMC10560260; doi:10.1038/s41467-023-41963-7)
Supplement: Supplementary file 3 — Description of Additional Supplementary Files [file 41467_2023_41963_MOESM3_ESM.pdf]

Description of Additional Supplementary Files

Title: Supplementary Data 1

Description: Association of BMI with plasma lipid species

Title: Supplementary Data 2

Description: Association of mBMIΔ with plasma lipid species

Title: Supplementary Data 3

Description: Weightings /beta coefficients from the optimum ridge and LASSO models

Title: Supplementary Data 4

Description: MRM transitions and conditions for examined lipid species

Title: Supplementary Data 5

Description: Coefficient of variation (CV) for the QC samples used in the AusDiab cohort
