# Supplementary material for: Metabolic phenotyping of BMI to characterize cardiometabolic risk: evidence from large population-based cohorts
Source: Nat Commun. 2023 Oct 7;14:6280. doi: 10.1038/s41467-023-41963-7 (PMC10560260; doi:10.1038/s41467-023-41963-7)
Supplement: Supplementary file 9 — Reporting Summary [file 41467_2023_41963_MOESM9_ESM.pdf]

nature portfolio

Corresponding author(s): Peter Meikle & Diana Magliano

Last updated by author(s): Aug 23, 2023

Reporting Summary

Nature Portfolio wishes to improve the reproducibility of the work that we publish. This form provides structure for consistency and transparency in reporting. For further information on Nature Portfolio policies, see our Editorial Policies and the Editorial Policy Checklist.

Statistics

For all statistical analyses, confirm that the following items are present in the figure legend, table legend, main text, or Methods section.

n/a	Confirmed	
☐	☒	The exact sample size (*n*) for each experimental group/condition, given as a discrete number and unit of measurement
☐	☒	A statement on whether measurements were taken from distinct samples or whether the same sample was measured repeatedly
☐	☒	The statistical test(s) used AND whether they are one- or two-sided<br>*Only common tests should be described solely by name; describe more complex techniques in the Methods section.*
☐	☒	A description of all covariates tested
☐	☒	A description of any assumptions or corrections, such as tests of normality and adjustment for multiple comparisons
☐	☒	A full description of the statistical parameters including central tendency (e.g. means) or other basic estimates (e.g. regression coefficient) AND variation (e.g. standard deviation) or associated estimates of uncertainty (e.g. confidence intervals)
☐	☒	For null hypothesis testing, the test statistic (e.g. *F*, *t*, *r*) with confidence intervals, effect sizes, degrees of freedom and *P* value noted<br>*Give P values as exact values whenever suitable.*
☒	☐	For Bayesian analysis, information on the choice of priors and Markov chain Monte Carlo settings
☒	☐	For hierarchical and complex designs, identification of the appropriate level for tests and full reporting of outcomes
☐	☒	Estimates of effect sizes (e.g. Cohen's *d*, Pearson's *r*), indicating how they were calculated

Our web collection on statistics for biologists contains articles on many of the points above.

Software and code

Policy information about availability of computer code

Data collection	Data consisted of epidemiological (anthropometric, clinical, lifestyle and diet)and lipidomic data. This study utilized the previously collected anthropometric, health behavior and clinical data as referenced in the methods section of the manuscript. A validated Food Frequency Questionnaire (FFQ) developed by the Anti-Cancer Council of Victoria (CCV) was used to collect data on dietary intake. Lipidomic data were generated using ESI-HPLC-MS/MS (6490 triple quadrapole, QQQ) instrument (Agilent Technologies, USA).
Data analysis	Mass Hunter (B.08.00, Agilent Technologies) software was used for HPLC-MS/MS data acquisition and analysis. Data analysis was performed as described in the method section of the manuscript. STATA v15 (StataCorp LP, Inc., Texas, USA) or R (version 3.6.1) were used to analyze the data. In particular R packages such as glmnet and caret were used for fitting ridge and LASSO models and ggplot2 for graphics. The glm package was used to fit linear regression models. Cox regression models were fitted to compute hazard ratios using age as the time scale using the survival R package (version 3.5.5).

For manuscripts utilizing custom algorithms or software that are central to the research but not yet described in published literature, software must be made available to editors and reviewers. We strongly encourage code deposition in a community repository (e.g. GitHub). See the Nature Portfolio guidelines for submitting code & software for further information.

Data

Policy information about availability of data

All manuscripts must include a data availability statement. This statement should provide the following information, where applicable:

- Accession codes, unique identifiers, or web links for publicly available datasets
- A description of any restrictions on data availability
- For clinical datasets or third party data, please ensure that the statement adheres to our policy

The Australian Diabetes, Obesity and Lifestyle Study (AusDiab) and Busselton Health Study (BHS) clinical, behavioral and demographic data collection is described in the methods section. Plasma (in the AusDiab) and serum(in the BHS cohort) lipidome were generated using ESI-HPLC-MS/MS (6490 triple quadrapole, QQQ) instrument (Agilent Technologies, USA). Because of the participant consent obtained as part of the recruitment process for the Australian Diabetes, Obesity and Lifestyle Study, it is not possible to make these data publicly available. Individual-level data will be made available upon reasonable written request to the study lead Professor Jonathan Shaw and the AusDiab Study Committee (Email: Jonathan.Shaw@baker.edu.au).

Research involving human participants, their data, or biological material

Policy information about studies with human participants or human data. See also policy information about sex, gender (identity/presentation), and sexual orientation and race, ethnicity and racism.

Reporting on sex and gender	We followed the sex and gender equity in research (SAGER) guideline and included the recommended information in our study. Sex information in both cohorts (AusDiab and BHS) were based on self-reported questionnaire and our study involves both sexes. We only have sex information in both cohorts and the number of participants from each sex is reported in Methods section, Table 1, in the Manuscript and Supplementary Table1, Supplementary Table2. Moreover, whenever possible sex-stratified analyses were reported including sex specific BMI models.
Reporting on race, ethnicity, or other socially relevant groupings	While 100% of the BHS participants were white, in the Ausdiab 94.7% were white/European ancestry and 5.3% were Asian/other ancestry (as classified by the participants). We have reported this information in the manuscript (Table 1) and in the Methods section.
Population characteristics	Basic characteristics of the cohorts is described below: 1) the Australian Diabetes, Obesity and Lifestyle Study , n= 10,339, mean (SD) age = 51.3 (14.3), mean (SD) BMI = 26.9(4.9) and 55% women. 2) Busselton Health Study, n= 4,492, mean (SD) age = 50.8 (17.4), mean (SD) BMI = 26.2 (4.2) and 56% women. A detailed description of the population characteristics is provided including information on sex composition (methods section, Supplementary Table1, Supplementary Table2)
Recruitment	This study did not directly involve the recruitment of participants. Recruitment for the AusDiab is extensively described in earlier paper (Dunstan et.al, 2002). For the BHS cohort, invitations to participate were sent to adults listed on earlier Electoral Registers (registration to vote is compulsory in Australia) for the Busselton district. Participants in the BHS cohort were all individuals who participated the 1994/95 survey of the long running epidemiological study. In 1994/95 all participants in the previous rounds of BHS study were invited to donate blood sample and complete assessments.
Ethics oversight	This study did not directly involve the recruitment of participants. We used datasets from the AusDiab biobank (project grant APP1101320) approved by the Alfred Human Research Ethics Committee, Melbourne, Australia (project approval number, 41/18) and the BHS cohort (informed consent obtained from all participants, and the study was approved by the University of Western Australia Human Research Ethics Committee [UWAHREC; approval number, 608/15]). Both studies were conducted in accordance with the ethical principles of the Declaration of Helsinki.

Note that full information on the approval of the study protocol must also be provided in the manuscript.

Field-specific reporting

Please select the one below that is the best fit for your research. If you are not sure, read the appropriate sections before making your selection.

☒ Life sciences ☐ Behavioural & social sciences ☐ Ecological, evolutionary & environmental sciences

For a reference copy of the document with all sections, see nature.com/documents/nr-reporting-summary-flat.pdf

Life sciences study design

All studies must disclose on these points even when the disclosure is negative.

Sample size	For the AusDiab, sample size was selected based on precision of estimates to identify a national diabetes prevalence of 7.0% (an estimation based on results of previous surveys, and the expectation that the diabetes rate had increased over time). Accounting for the clustering of the survey design, a sample size of 11,247 (~1500 per state) was predicted to provide 95% confidence intervals of 6.2–7.8, around a diabetes estimate of 7.0%. The details on sampling and sample size had been described in earlier paper (Dunstan et.al, 2002). For lipidomics a total of of 10,339 participants were included after excluding some individuals (on the basis of criteria listed below). This level of precision was regarded as acceptable, and the sample size was considered highly powered. In the BHS a total of 4492 participants who were recruited from earlier Electoral Registers (registration to vote is compulsory in Australia) for the Busselton district and those completed questionnaire and biochemical tests in 1994/95 were employed.

Data exclusions	We utilized all baseline fasting plasma samples from the AusDiab cohort (n = 10,339) (Table 1) after excluding samples from pregnant women (n =21), those with missing data (n= 277), technical reasons (n=19) or whose fasting plasma samples were unavailable (n=591). No data was excluded for the BHS 1994/95 study at baseline.
Replication	The BMI models were generated in a 10 fold cross validation framework splitting the discovery dataset into training and testing datasets and evaluated all the models with the hold out testing set to confirm the robustness and avoid over fitting. Moreover, we utilized the BHS cohort to validate the the findings observed in the AusDiab including the BMI model and the mBMI score-disease associations.
Randomization	The participant recruitment and data collection for both the AusDiab and BHS cohorts were conducted before the current study. It is important to note that the objectives of the original studies and the current study differ. During data analysis, appropriate adjustments were made by including covariates such as age, sex, and BMI in the models. Additional confounders were also taken into account as deemed necessary for the analysis. Samples were randomized prior lipidomic analysis
Blinding	Since data collection and data analysis were carried out independently by different researchers, no additional blinding measures were implemented. Data analysts, including statisticians and bioinformaticians, work with de-identified data, ensuring that they are unaware of any specific laboratory, clinical, or lipidomics information. All statistical analyses and graphics are conducted using computer software without any manual interventions.

Reporting for specific materials, systems and methods

We require information from authors about some types of materials, experimental systems and methods used in many studies. Here, indicate whether each material, system or method listed is relevant to your study. If you are not sure if a list item applies to your research, read the appropriate section before selecting a response.

Materials & experimental systems

n/a	Involved in the study
☒	☐ Antibodies
☒	☐ Eukaryotic cell lines
☒	☐ Palaeontology and archaeology
☒	☐ Animals and other organisms
☒	☐ Clinical data
☒	☐ Dual use research of concern
☒	☐ Plants

Methods

n/a	Involved in the study
☒	☐ ChIP-seq
☒	☐ Flow cytometry
☒	☐ MRI-based neuroimaging
